# Supplementary material for: Residual risk of cardiovascular complications in statin-using patients with type 2 diabetes: the Taiwan Diabetes Registry Study
Source: Lipids Health Dis. 2024 Jan 23;23:24. doi: 10.1186/s12944-023-02001-z (PMC10804647; doi:10.1186/s12944-023-02001-z)
Supplement: Supplementary file 1 — Supplementary Material 1 [file 12944_2023_2001_MOESM1_ESM.pdf]

Residual Risk of Cardiovascular Complications in Statin-Using Patients with Type 2 Diabetes: The Taiwan Diabetes Registry Study

By CS KUO

WORD COUNT 3303

TIME SUBMITTED 19-DEC-2023 04:16PM

PAPER ID 105600270

Abstract

Background The residual risks of atherosclerotic cardiovascular disease in statin-treated patients with diabetes remain unclear. This study was conducted to identify factors associated with these residual risks in patients with no prior vascular event.

Methods Data on 683 statin-using patients with type 2 diabetes mellitus (T2DM) from the Taiwan Diabetes Registry were used in this study. Patients aged <25 or >65 years at the time of diabetes diagnosis and those with diabetes durations ≥ 20 years were excluded. The United Kingdom Prospective Diabetes Study risk engine (version 2.01; https://www.dtu.ox.ac.uk/riskengine/) was used to calculate 10-year residual nonfatal and fatal coronary heart disease (CHD) and stroke risks. Associations of these risks with physical and biochemical variables, including medication use and comorbidity, were examined.

Results The 10-year risks of nonfatal CHD in oral anti-diabetic drug (OAD), insulin and OAD plus insulin groups were 11.8%, 16.0%, and 16.8%, respectively. The 10-year risks of nonfatal stroke in OAD, insulin and OAD plus insulin groups were 3.0%, 3.4%, and 4.3%, respectively. In the multivariate model, we determined that chronic kidney disease (CKD), neuropathy, insulin use, calcium-channel blocker (CCB) use, higher body mass indices (BMI), low-density lipoprotein (LDL), fasting glucose, log-triglyceride (TG), and log–alanine transaminase (ALT) levels were associated with an increased CHD risk. The residual risk of stroke was associated with CKD, neuropathy, CCB use, and lower LDL cholesterol levels, higher BMI and diastolic blood pressure.

Conclusion This study indicated that insulin was probably a residual risk factor of CHD but not stroke, and that there was a possible presence of obesity paradox in patients with T2DM on statin therapy. In addition to lowering TG and normalizing fasting glucose levels, lower LDL cholesterol level is better for reduction of risk of CHD on statin therapy. On the other hand, lower LDL cholesterol level could potentially be related to higher risk of stroke among

populations receiving statin therapy. Our findings suggest potential therapeutic targets for residual cardiovascular risk reduction in patients with T2DM on statin therapy.

Keywords: Diabetes, Residual risk, Statin

Background

The increasing prevalence of type 2 diabetes mellitus (T2DM) is a significant global healthcare concern[1]. According to the UK Prospective Diabetes Study (UKPDS), nearly half of the deaths that occur within a decade of being diagnosed with DM are attributable to cardiovascular disease (CVD)[2]. Overall mortality due to vascular complications of DM increased by more than 30% between 2000 and 2016[3].

Statins play a large role in the reduction of the low-density lipoprotein (LDL) cholesterol level, a main objective in the management of increased CVD risk[4]. A meta-analysis revealed that the occurrence of major vascular events among diabetic patients taking statins declined significantly (by 21%) with every 1 mmol/L decrease in the LDL cholesterol level, but that about 14% of these patients experienced cardiovascular events during a 5-year period[5]. According to the Multi-Ethnic Study of Atherosclerosis[6], this residual atherosclerotic cardiovascular disease (ASCVD) risk in adults under statin treatment without prior ASCVD was associated with older age, male sex, previous or current smoking, higher systolic blood pressure (SBP), antihypertensive medication use, DM, and lower high-density lipoprotein (HDL) cholesterol levels. However, the residual risk has not been investigated thoroughly in patients withT2DM and no prior ASCVD. Thus, this study was conducted to identify parameters associated with the residual ASCVD risk in patients with T2DM and no prior ASCVD on statin therapy.

Methods

Data Sources and Subjects

This cross-sectional study was performed with data from the prospective Taiwan Diabetes Registry Study (TDRS) of the Diabetes Association of the Republic of China (Taiwan)[7]. Data from patients with recently diagnosed (<6 months) T2DM, obtained by

interviews with certified diabetes educators at the time of TDRS enrollment. Ninety-five primary care clinics and hospitals participated in the TDRS, and enrollment began in October 2015. The study was approved by the institutional review board of Taipei Veterans General Hospital (2015-08-003AC) and Taiwan's Joint Institutional Review Board (14-S- 012) and conducted in accordance with the Declaration of Helsinki. Participants in all studies provided written informed consent.

Study Variables

We extracted data on patients' age, sex, smoking status, DM duration, history of hepatitis B or C infection, body mass index (BMI), waist circumference, SBP and diastolic blood pressure (DBP), heart rate (HR), complications of DM [cerebrovascular events, coronary and peripheral artery disease, retinopathy, neuropathy, and chronic kidney disease (CKD)], history of comorbidities, blood and urine laboratory findings, and medication use. Cerebrovascular events encompassed hemorrhagic and ischemic stroke. Peripheral artery diseases were those presenting with intermittent claudication, foot ulceration, impalpable pulsation at the dorsalis pedis or posterior tibial artery, and/or amputation history. Retinopathies (proliferative and nonproliferative) were conditions diagnosed with maculopathy and/or unilateral blindness. Neuropathies were defined by abnormal monofilament or vibration test results. CKD was defined by estimated glomerular filtration rate (eGFR) < 60 mL/min/1.73 m^2 (determined using the Cockcroft-Gault equation) and proteinuria (at least trace results of a urine dipstick test or urine albumin:creatinine ratio ≥ 30). Medication use encompassed statins, antihypertensives [e.g., angiotensin receptor blockers (ARBs), angiotensin-converting enzyme inhibitors (ACEis), calcium channel blockers (CCBs), diuretics, and alpha blockers], insulin, and oral antidiabetic drugs (OADs).

Inclusion and Exclusion Criteria

Data on patients using statins were included in the analysis. To select a population to which the UKPDS risk engine could be applied, we excluded patients with non-T2DM, age <25 or >65 years at the time of diabetes diagnosis, and diabetes duration ≥ 20 years. We further excluded patients who controlled their diabetes solely with lifestyle modifications and did not use insulin or OADs; those with previous CVD (coronary and peripheral artery disease and stroke); and those with serious life-threatening illnesses such as heart failure, renal failure (hemodialysis, peritoneal dialysis, and kidney transplantation), malignancies (e.g., lung, liver, colorectal, breast, endometrial, cervical, stomach, pancreatic, urinary tract, prostate, thyroid, head and neck, and skin cancers), and arrhythmia. Due to the computation of missing cholesterol data, patients with missing HDL cholesterol and total or LDL cholesterol values and those with triglyceride (TG) levels > 500 mg/dL were excluded.

Group Allocation and Outcomes

The patients were allocated to OAD, insulin, and OAD plus insulin groups. The 10-year risks of nonfatal and fatal coronary heart disease (CHD) and stroke were calculated using the UKPDS risk engine (version 2.01; https://www.dtu.ox.ac.uk/riskengine/) and compared among groups. We also evaluated the ability of the study variables to predict these risks.

Statistical Analysis

Categorical variables are expressed as numbers and percentages and were analyzed using the chi-squared test. Continuous variables are expressed as means ± standard deviations and were examined using analysis of variance, followed by Sheffe's multiple comparison post hoc test. In risk calculation using the UKPDS risk engine, 95% confidence intervals were calculated and missing fasting glucose, creatinine, and eGFR data were

imputed using multiple imputation by chained equations with the R studio cloud[8]. Missing cholesterol data were computed using the equation total cholesterol = TG/5 + HDL cholesterol + LDL cholesterol. Patients with computed HDL cholesterol values < 0 mg/dL and those with risk values exhibiting error due to small HDL cholesterol values were excluded from further analysis.

To evaluate the ability of physical and biochemical variables to predict the 10-year risks of nonfatal and fatal CHD and stroke, we first constructed univariate linear regression models. Serum TG and alanine transaminase (ALT) values were also analyzed as natural logarithms (logTG and logALT). Variables with P values < 0.05 in the univariate analysis and those with the smallest P values between TG/logTG, ALT/logALT, and serum creatinine/eGFR were entered into multivariate regression models. The statistical analyses were conducted with SPSS software (version 23.0 for Windows; IBM Corporation, Armonk, NY, USA) and a significance level of $P < 0.05$.

Results

Patient Characteristics

Of 1147 diabetic patients using statins identified, data from 683 were included in the final analysis (Figure 1). The OAD, insulin, and OAD plus insulin groups contained 576, 30, and 77 patients, respectively.

The patients' characteristics are presented in Table 1. Subjects using OAD alone (50.7 ± 9.2 years old) are older in diabetes onset compared to those under therapy of both OAD and insulin (48.0 ± 9.4 years old) (p=0.021). Subjects using OADs and insulin (5.6 ± 7.2 years) had longer DM durations than did those using insulin alone (2.5 ± 5.3 years) (p=0.038) or OADs alone (2.3 ± 4.8 years) (p<0.001). The glycated hemoglobin (HbA1c) concentration was higher in the insulin alone group (9.6 ± 2.8) (p=0.004) and insulin-OAD combination group

(9.1 ± 2.5) (p=0.002) compared to the OADs alone group (8.1 ± 2.2). Subjects using both OADs and insulin (16/77, 20.8%) were more likely to develop CKD than were those using OADs alone (58/576, 10.1%)) (p=0.011).

The 10-year risks of nonfatal CHD in OAD, insulin and OAD plus insulin groups were 11.8%, 16.0%, and 16.8%, respectively. The 10-year risks of fatal CHD in OAD, insulin and OAD plus insulin groups were 6.4%, 9.6%, and 10.9%, respectively. The 10-year risks of nonfatal stroke in OAD, insulin and OAD plus insulin groups were 3.0%, 3.4%, and 4.3%, respectively. The 10-year risks of fatal stroke in OAD, insulin and OAD plus insulin groups were 0.3%, 0.4%, and 0.4%, respectively. Patients who used both insulin and OAD (16.8%, 95% CI 12.5~21.1%) had higher 10-year risk of nonfatal (p=0.025) CHD, compared to those under OAD monotherapy (11.8%, 95% CI 10.6~13.0%). Similarly, patients who used both insulin and OAD (10.9%, 95% CI 7.5~14.3%) had higher 10-year risk of fatal (p=0.002) CHD, compared to those under OAD monotherapy (6.4%, 95% CI 5.6~7.1%). On the contrary, there was no significant difference in 10-year risk of nonfatal and fatal CHD between those under insulin alone and OAD alone, or between insulin alone and combination therapy. Patients who used both insulin and OAD (4.3%, 95% CI 3.2~5.5%) had higher 10-year risk of nonfatal stroke (p=0.043), compared to those under OAD monotherapy (3.0%, 95% CI 2.7~3.4%). Nevertheless, there were no significant difference between either two groups in the 10-year risk of fatal stroke. (Figure 2).

Factors Associated with the Nonfatal and Fatal CHD Risks

Table 2 showed clinical and laboratory factors and their standardized coefficients (β) that predict the 10-year risks of nonfatal and fatal CHD and stroke.

In the univariate model, patients having diabetic complications including neuropathy and CKD, those with higher logTG, LDL cholesterol, fasting glucose, and those under

medications such as CCB and insulin were associated with significantly greater risks of nonfatal CHD (Table 2). Similarly, greater risks of fatal CHD were associated with neuropathy and CKD; higher logTG, LDL cholesterol, fasting glucose, and the use of CCB and insulin. Lower BMI and lower logALT were associated with a greater nonfatal CHD risk. Similarly, greater risks of fatal CHD were associated with lower BMI and lower logALT (Table 2).

In the multivariate model, lower BMI and lower logALT level; higher logTG, LDL cholesterol, fasting glucose, CCB and insulin use were associated independently with a greater nonfatal CHD risk (Table 2). Similarly, in the multivariate model, greater risk of fatal CHD was independently associated with lower BMI and lower logALT level; higher logTG, LDL cholesterol, fasting glucose, CCB and insulin use.

Factors Associated with Nonfatal and Fatal Stroke Risks

In the univariate model, diabetic patients complicated with CKD and neuropathy, having lower DBP, BMI, LDL cholesterol, and logALT, and using medications including ARB, CCB, diuretics were at an elevated nonfatal 10-year stroke risk. Among the above-mentioned factors, lower DBP, BMI, and LDL cholesterol, and the use of CCB remained significant in the multivariate analysis.

In the univariate model, an increased residual risk of fatal stroke was associated with diabetic complications including retinopathy, neuropathy, CKD. In addition, patients with lower BMI, LDL cholesterol, and logALT and those using ARB, CCB, and diuretics were associated with higher risks of fatal stroke. In the multivariate regression model, independent risk factors of fatal stroke included lower BMI, logALT, and the use of CCB.

Discussion

In this study, we identified factors affecting the residual risks of CHD and stroke in patients

with diabetes under statin treatment. In addition to CKD, neuropathy, and insulin and CCB use, we determined that higher LDL, fasting glucose, and logTG levels were associated with an increased CHD risk. We advance knowledge in this area by further showing that higher BMI and logALT values were associated with a reduced risk of CHD in this population. The residual risk of stroke was associated with CKD, neuropathy, CCB use, and low LDL cholesterol levels, lower BMI and lower DBP in this study. The reduction of both residual risks of CHD and stroke with higher BMI suggests that the obesity paradox can be applied to patients with T2DM on statin therapy.

Obesity is known to increase the CVD risk in the general population[9], but to prolong survival in patients with CVD[10]. The obesity paradox has also been reported to apply to patients with T2DM, possibly because genetically susceptible individuals develop T2DM at lower BMIs and have elevated risks of other diseases and complications, and thus poor prognoses[11,12]. Another explanation is that confounding factors have not been addressed properly in previous research, potentially leading to the underestimation of the effect of the BMI on the mortality risk[13]. The underlying mechanism of obesity paradox in this study needs further research to be clarified.

Interestingly, the role of LDL cholesterol in residual risks of CHD showed different from in that of stroke in this study. There is expansion of evidence in “lower is better” principle of LDL cholesterol management for prevention of CHD[14,15]. In concordance with the previous findings, we found that lower LDL cholesterol levels were associated with the reduced risk of CHD among populations receiving statin therapy. Furthermore, previous reports demonstrated that lipid-lowering therapy reduced risks of ischemic stroke but increased risks of hemorrhagic stroke[16]. In this study, lower LDL cholesterol levels associated with higher risk of stroke. One possible explanation might be increased risks of hemorrhagic stroke. The adequate LDL cholesterol target in patients with T2DM on statin therapy would

need individual consideration of prevention CHD or stroke.

We demonstrated that higher LDL cholesterol and lower logALT levels were residual risk factors of CHD. Consistently, in the Treating to New Targets study, higher atorvastatin dosages were associated with lower LDL cholesterol levels, higher ALT levels, and fewer first major cardiovascular events in patients with diabetes[17]. The Pravastatin or Atorvastatin Evaluation and Infection Therapy trial also demonstrated that the statin dose was related positively to the incidence of transaminitis[18]. The possible explanation is that higher ALT levels associated with higher statin dose and lower CHD complications.

Insulin use was a residual risk factor of CHD but not stroke in this study. The cardiovascular outcomes of insulin therapy remain controversial. Findings from the large Action to Control Cardiovascular Risk in Diabetes[19] and Outcome Reduction with Initial Glargine Intervention[20] randomized controlled trials suggest that insulin has a neutral effect on cardiovascular diseases, whereas some observational studies[21,22] have revealed that it substantially increases cardiovascular risks. This discrepancy can be explained in part by confounding by factors such as diabetes severity and renal impairment[23]. However, our multivariate analyses were adjusted for these factors. Another possible explanation is that insulin users in our population were younger at diabetes onset and had higher HbA1c concentrations (independent risk factors for adverse cardiovascular events[24]) relative to non-users.

In our adjusted analyses, higher DBP was associated with better stroke outcomes. This finding suggests that the CVD risk increases with the pulse pressure, consistent with the demonstration in a meta-analysis that CVD-related mortality increases by nearly 20% with a 10-mm Hg increase in the pulse pressure[25]. The increased CVD risk with low DBP has been attributed to decreased perfusion to vital organs and the parallel increase in SBP with age-related arterial stiffening[26].

Our result suggested that logTG was a residual risk factor for CHD. This finding is supported by a previous retrospective study showing a strong association between TG levels greater than 150 mg/dL and initial major adverse cardiovascular events among statin-treated diabetic patients with LDL cholesterol levels < 100 mg/dL[27]. Hypertriglyceridemia-related subclinical atherosclerosis and vascular inflammation may contribute to atherosclerotic plaque development and subsequent cardiovascular events independently of the LDL cholesterol concentration[27,28]. These findings suggest that the reduction of the TG level is a potential target for the reduction of the residual risks of CHD events in diabetic patients with LDL concentrations falling within guideline recommendations.

In agreement with previous findings, this study showed that CKD is a risk factor for poor cardiovascular outcomes in patients with diabetes under statin treatment. An analysis of data from the Third National Health and Nutrition Examination Survey showed that CKD was associated independently with an increased cardiovascular mortality risk among patients with diabetes[29]. Statins were found to protect against cardiovascular complications in patients with diabetes and CKD in one study[30], but to have reduced efficacy with deteriorating renal function in a meta-analysis[31]. These findings may be explained by the occurrence of vascular calcification in advanced CKD[32].

Peripheral neuropathy was a residual risk factor for CHD and stroke in this study. In diabetic patients, neuropathy shares risk factors with cardiovascular complications[33]. Peripheral neuropathy independently predicts initial CVD in patients with T2DM[34]. The association of peripheral neuropathy with cardiovascular events likely reflects the involvement of common pathways, including systemic inflammation[35] and lipid dysmetabolism[36], which may be reversed by statin therapy[37]. The cross-sectional and longitudinal Fremantle Diabetes Study showed that statins prevented diabetic neuropathy[38], potentially due to their lipid-lowering, endothelial cell–activating, and anti-inflammatory

effects. However, other pathogenetic mechanisms, such as the chronic hyperglycemia-facilitated deposition of advanced glycation end products in nerves[39] and vessels[40], which leads to diabetic neuropathy and cardiovascular events, are not related clearly to statin use.

Current American Diabetes Association guidelines recommend an ACEi or ARB as the first-line medication for diabetes, especially in patients with proteinuria, and the addition of CCB as a second-line medication[41]. The observed association of CCB use with increased CHD and stroke risks[42] could be explained by polypharmacy-related poor cardiovascular drug compliance and the subsequent increased risk of adverse outcomes[43].

This study has several limitations. Its cross-sectional design prevented the establishment of causal relationships; prospective longitudinal studies are needed to investigate the causal relationships between residual risk factors and cardiovascular outcomes. In addition, the types, dosages, and use durations of statins and anti-diabetic medications were not included in the study data and thus were not examined. Variation in the underlying mechanisms, potency, and side effects of medications in the same class may influence patient adherence, laboratory findings, and the endpoints examined in this study. Furthermore, this study used BMI to infer “obesity paradox”. However, BMI is not synonymous with excess adiposity, measured by body fat percentage. Further research may include measurements of body composition and their distribution for elucidation of obesity paradox. Additionally, data from the present study were insufficient to differentiate between ischemic and hemorrhagic strokes in the analysis.

Conclusions

Based on the findings of this study, insulin may possibly increase risk of of CHD but not stroke in statin-treated patients with T2DM. We suggest the use of higher statin doses to lower LDL cholesterol levels, even with ALT elevation, to further reduce CHD risks in patients with T2DM. On the other hand, lower LDL cholesterol level and lower DBP related to higher

risk of stroke among populations receiving statin therapy. Therefore, in patients with T2DM on statin therapy, a higher LDL cholesterol target may possibly be acceptable in those with lower DBP. Under statin therapy and with the control of other known risk factors, residual cardiovascular risks may be lower in obese than in non-obese subjects with T2DM. CKD, neuropathy, and higher TG levels were also residual risk factors in this study, as was the use of CCBs, possibly representing poly-antihypertensive agent use.

Figure legends

Figure 1. Flowchart of study subject selection process

Figure 2. Residual risk of nonfatal, fatal coronary heart disease and stroke in oral antidiabetic drug (OAD) monotherapy group, insulin monotherapy group, and OAD+Insulin dual therapy group

Residual Risk of Cardiovascular Complications in Statin-Using Patients with Type 2 Diabetes: The Taiwan Diabetes Registry Study

ORIGINALITY REPORT

9%
SIMILARITY INDEX

PRIMARY SOURCES

	Source	Match
1	www.science.gov<br>Internet	39 words — 1%
2	link.springer.com<br>Internet	37 words — 1%
3	"Handbook of nutrition in heart health", Brill, 2017<br>Crossref	26 words — 1%
4	ecommons.aku.edu<br>Internet	25 words — 1%
5	bmjopen.bmj.com<br>Internet	16 words — < 1%
6	Eberhard Wille, Jürgen Scholze, Eduardo Alegria, Claudio Ferri et al. "Modelling the costs of care of hypertension in patients with metabolic syndrome and its consequences, in Germany, Spain and Italy", The European Journal of Health Economics, 2010<br>Crossref	15 words — < 1%
7	bmcpsychiatry.biomedcentral.com<br>Internet	15 words — < 1%

www.diva-portal.org

8 Internet 15 words — < 1%

9 www.spandidos-publications.com
Internet 15 words — < 1%

10 Xue Sun, Jie He, Xiao-Li Ji, Yi-Ming Zhao et al. "Association of Chronic Kidney Disease with Coronary Heart Disease and Stroke Risks in Patients with Type 2 Diabetes Mellitus", Chinese Medical Journal, 2017
Crossref 14 words — < 1%

11 pubs.sciepub.com
Internet 14 words — < 1%

12 pure.rug.nl
Internet 12 words — < 1%

13 globalizationandhealth.biomedcentral.com
Internet 11 words — < 1%

14 ir.ymlib.yonsei.ac.kr
Internet 11 words — < 1%

15 Masson, Patricia R.. "Factors Influencing Blood Pressure Control Among Hypertensive Adults 35 to 64 Years of Age at Risk for Stroke", University of Massachusetts Lowell, 2023
ProQuest 9 words — < 1%

16 arrhythmia.biomedcentral.com
Internet 9 words — < 1%

17 ndltd.ncl.edu.tw
Internet 9 words — < 1%

18 www.frontiersin.org
Internet

9 words — < 1%

19 www.researchgate.net
Internet
9 words — < 1%

20 www.researchsquare.com
Internet
9 words — < 1%

21 R. R. Holman. "Atorvastatin in Factorial with Omega-3 EE90 Risk Reduction in Diabetes (AFORRD): a randomised controlled trial", Diabetologia, 01/2009
Crossref
8 words — < 1%

22 Bianchi, C.. "Non-traditional markers of atherosclerosis potentiate the risk of coronary heart disease in patients with type 2 diabetes and metabolic syndrome", Nutrition, Metabolism and Cardiovascular Diseases, 200801
Crossref
7 words — < 1%

23 Roberto B. Vargas, Carol M. Mangione, Steven Asch, Joan Keesey, Mayde Rosen, Matthias Schonlau, Emmett B. Keeler. "Can a Chronic Care Model Collaborative Reduce Heart Disease Risk in Patients with Diabetes?", Journal of General Internal Medicine, 2007
Crossref
6 words — < 1%

EXCLUDE QUOTES	OFF	EXCLUDE SOURCES	OFF
EXCLUDE BIBLIOGRAPHY	OFF	EXCLUDE MATCHES	OFF
