# Supplementary material for: Residual risk of cardiovascular complications in statin-using patients with type 2 diabetes: the Taiwan Diabetes Registry Study
Source: Lipids Health Dis. 2024 Jan 23;23:24. doi: 10.1186/s12944-023-02001-z (PMC10804647; doi:10.1186/s12944-023-02001-z)
Supplement: Supplementary file 2 — Supplementary Material 2 [file 12944_2023_2001_MOESM2_ESM.pdf]

WRITE SCIENCE RIGHT
5923 Vizzi Ct. • Las Vegas, NV 89131 USA • www.WriteScienceRight.com

December 19th, 2023

Re: Certification of manuscript editing

To Whom It May Concern:
The purpose of this letter is to certify that **Chin-Sung Kuo** received professional English language editing services from our company, Write Science RightTM, for a paper titled **"Residual Risk of Cardiovascular Complications in Statin-Using Patients with Type 2 Diabetes: The Taiwan Diabetes Registry Study"** (order 230625-1). The work was completed by a native English-speaking American biomedical scientist and professional scientific editor.

All grammatical/syntactical errors and awkward verbiage have been revised to make the paper ready to submit for publication review. Assuming the author has accepted our advice and that the paper has not been modified subsequently, it should be ready to review. Feel free to contact me if you have any questions.

Best regards,

Ann Power Smith, PhD
Write Science Right, CSO
info@writescienceright.com
(949)929-2196
